# Сочетание феноменов макро-ТТГ и макропролактинемии у пациента с аутоиммунным тиреоидитом и витилиго

**DOI:** 10.14341/probl13390

**Published:** 2024-11-05

**Authors:** Д. В. Сазонова, М. А. Перепелова, А. С. Шутова, Л. В. Никанкина, Г. С. Колесникова, Е. А. Пигарова, Л. К. Дзеранова

**Affiliations:** Национальный медицинский исследовательский центр эндокринологии; Национальный медицинский исследовательский центр эндокринологии; Национальный медицинский исследовательский центр эндокринологии; Национальный медицинский исследовательский центр эндокринологии; Национальный медицинский исследовательский центр эндокринологии; Национальный медицинский исследовательский центр эндокринологии; Национальный медицинский исследовательский центр эндокринологии

**Keywords:** макротиротропин (макро-ТТГ), макропролактин, макропролактинемия, макротиротропинемия (макро-ТТГ-емия), аутоиммунный тиреоидит, витилиго

## Abstract

Лабораторные методы диагностики являются основными инструментами в практике врача любой специальности, в том числе эндокринолога. Были определены факторы, способные изменять концентрацию биологически активной фракции исследуемого вещества, в последующем затрудняющие интерпретацию лабораторных результатов и принятие верного клинического решения. В литературе представлено описание множества циркулирующих аутоантител, участвующих в связывании с гормонами гипофиза (пролактином (ПРЛ), тиреотропным (ТТГ), соматотропным, лютеинизирующим, фолликулостимулирующим и адренокортикотропным гормонами), гипоталамуса (вазопрессином и окситоцином), поджелудочной железы (инсулином и глюкагоном), околощитовидных желез (паратиреоидным гормоном), а также с гормонами щитовидной железы. Как правило, образуемые макромолекулы приводят к повышению лабораторных показателей, в которых превалирующая фракция гормона не обладает биологической активностью, что и определяет основную проблему данного феномена. К наиболее часто встречаемым вариантам относят иммунные комплексы с ПРЛ и ТТГ, вызывающие феномены макропролактинемии и макротиротропинемии (макро-ТТГ-­емии/макро-ТТГ) соответственно. Большинство лабораторных тест-систем, используемых в клинической практике, способны определять лишь общее количество ПРЛ и ТТГ.

В представленном клиническом наблюдении описано сочетание феноменов макро-ТТГ и макропролактинемии у пациента с аутоиммунным тиреоидитом (АИТ) и витилиго.

## Abstract (en)

Laboratory diagnostic methods are the main tools in the practice of a doctor of any specialty, including an endocrinologist. Factors were identified that could change the concentration of the biologically active fraction of the test substance, subsequently complicating the interpretation of laboratory results and making the right clinical decision. The literature describes a variety of circulating autoantibodies involved in binding to pituitary hormones (prolactin (PRL), thyroid-stimulating hormone (TSH), growth hormone, luteinizing, follicle-stimulating, and adrenocorticotropic hormones), hypothalamus (vasopressin and oxytocin), pancreas (insulin and glucagon) , parathyroid glands (parathyroid hormone), as well as with thyroid hormones. As a rule, the resulting macromolecules lead to an increase in laboratory parameters, in which the prevailing fraction of the hormone does not have biological activity, which determines the main problem of this phenomenon. The most common variants include immune complexes with PRL and TSH, causing the phenomena of macroprolactinemia and macrothyrotropinemia (macro-TSH-emia/macro-TSH), respectively. Most laboratory test systems used in clinical practice are capable of determining only the total amount of PRL and TSH. The presented clinical observation describes a combination of the phenomena of macro-TSH and macroprolactinemia in a patient with autoimmune thyroiditis (AIT) and vitiligo.

## АКТУАЛЬНОСТЬ

В настоящее время под лабораторным феноменом макропролактинемии подразумевается преобладание высокомолекулярной фракции пролактина (более 60% от общего пролактина) при гиперпролактинемии [10–12]. В свою очередь, макропролактин является иммунным антиген-антительным комплексом, состоящим из мономерного пролактина (23 кДа) и иммуноглобулина G (IgG). В связи со своей большой молекулярной массой (более 150 кДа) данная фракция получила второе название «big-big» пролактин. Впервые о подобном феномене сообщили Whittaker P. et al. [13] в 1981 г., описав пациентку с отсутствием характерной клинической картины синдрома гиперпролактинемии (аменорея, галакторея, бесплодие) на фоне лабораторно подтвержденного повышения пролактина. Встречаемость гиперпролактинемии в общей популяции составляет порядка от 0,15 до 1,6%, среди которой на феномен макропролактинемии приходится около 25% (5–35%) случаев [14–16].

Вторым по распространенности иммунным комплексом является макротиротропин (макро-ТТГ). Как правило, феномен макротиротропинемии (макро-ТТГ-емии, макро-ТТГ) можно заподозрить при персистирующем уровне ТТГ (более 10–20 мМЕ/л) в ответ на лечение левотироксином натрия и в отсутствие вторичных причин его повышения [17][18]. Макро-ТТГ по аналогии с макропролактином представляет собой форму ТТГ, состоящую из комплекса мономерного ТТГ и IgG. Мономерная фракция ТТГ имеет молекулярную массу 28 кДа, в свою очередь макро-ТТГ, как и макропролактин, — более 150 кДа. Макро-ТТГ-емия является очень редким лабораторным феноменом, чья распространенность варьирует от 0,6% до 1,6% [12–14].

Патогенез образования данных макромолекул до сих пор остается до конца не изученным. Однако в ходе множества исследований продемонстрирован аутоиммунный генез образования макроизоформ, состоящих, как правило, из аутоантитела (IgG) к гормону, и мономерной фракции самого гормона.

В качестве «золотого стандарта» для фракционирования и количественного определения различных форм гормонов принят метод гель-фильтрационной хроматографии [19][20]. Однако данный вид лабораторного исследования требует значительных материальных, технических и временных затрат, в связи с чем не получил широкого распространения в клинической практике. Более подходящим для скрининговой диагностики лабораторных феноменов стало использование метода преципитация с полиэтиленгликолем (ПЭГ), базирующегося на иммуноопосредованной дифференцировке высокомолекулярных форм гормонов. В свою очередь к недостаткам ПЭГ относят значимые потери мономерной фракции гормона в преципитате, достигающие порядка 20–25% за счет «матричного» эффекта сыворотки крови [21].

В данной публикации представлено описание клинического случая пациента с первичным гипотиреозом в исходе АИТ, витилиго в сочетании с феноменами макро-ТТГ и макропролактинемии.

## ОПИСАНИЕ СЛУЧАЯ

Впервые в возрасте 25 лет у пациента А. после сильного эмоционального стресса появились жалобы на периодические отеки лица, слабость и утомляемость. В 27 лет самостоятельно обратился к эндокринологу, в ходе обследования выявлено повышение уровня ТТГ до 159 мМЕ/л при свТ4 9,9 пмоль/л (9–19), что было расценено как манифестный гипотиреоз и инициирована терапия левотироксином натрия в дозе 150 мкг/сут при массе тела пациента 72 кг (ИМТ=24,2 кг/м2). В последующем уровень ТТГ регулярно не определялся.

В динамике через 2 года при соблюдении правил сдачи гормонального анализа крови показатель ТТГ составил 0,42 мМЕ/л на фоне значимо высокого уровня свТ4 — 33,1 пмоль/л, что соответствовало ятрогенному тиреотоксикозу, в связи с чем доза левотироксина натрия уменьшена до 100 мкг/сутки. На фоне регулярного приема в течение четырех лет левотироксина натрия в дозе 100 мкг/сут сохранялся стабильно высокий показатель ТТГ — 94,2 мМЕ/л со сниженным уровнем свТ4 до 8,4 пмоль/л, низконормальным свТ3 — 2,6 пмоль/л (2,6–5,7) и положительным результатом АТ к ТПО — 210 МЕ/мл.

Также у пациента при первичном обращении к эндокринологу отмечалось повышение общего пролактина до 842 мЕд/мл (66–436), а по данным МРТ головного мозга с к/у выявлена микроаденома гипофиза размерами 0,43х0,38 см, в связи с чем эндокринологом по м/ж инициирован прием каберголина в дозе 0,5 мг 1 раз в неделю. Сочетанная гормональная активность аденомы была исключена: ИФР-1 267,2 нг/мл. При динамическом контроле на фоне нерегулярного приема агониста дофаминовых рецепторов уровень общего пролактина составил 19,94 мкМЕ/л (86–324). Через 4 года уровень пролактина — 27,65 мкМЕ/л, пациент самостоятельно отменил прием каберголина. При контрольной МРТ головного мозга с к/у в 30 лет подтверждена умеренная отрицательная динамика в виде увеличения размеров аденомы гипофиза до 0,9х0,7 см.

Ухудшение самочувствия пациент А. отметил в 33 года, когда появились выраженные отеки на лице, сопровождающиеся чувством «кома в горле» и трудностями при дыхании (со слов, данное состояние купировалось в/м введением преднизолона), появились периодические высыпания на теле по типу крапивницы с разрешением в виде пигментных пятен (без эффекта от приема антигистаминных препаратов), предположительно, на фоне приема пациентом витамина С (до 5000 мг в сутки). При лабораторном исследовании выявлено повышение уровня С-реактивного белка до 10,37 мг/л (норма до 5) при референсных показателях ревматоидного фактора — 3,49 МЕ/мл (до 14) и иммуноглобулина Е — 22 МЕ/мл (до 87), госпитализирован в отделение нейроэндокринологии в отделение нейроэндокринологии ГНЦ РФ ФГБУ «НМИЦ эндокринологии» Минздрава России.

При первичном осмотре обращали на себя внимание изменения цвета кожи по типу витилиго в области запястья на тыльной стороне правой и левой рук. При поступлении телосложение нормостенического типа, масса тела — 79 кг, рост — 172,7 см (ИМТ=26,5 кг/м2). Распределение подкожной жировой клетчатки равномерное, отеки не определялись.

В ходе госпитализации по результатам гормонального исследования крови было выявлено повышение уровня ТТГ до 58,88 мМЕ/л на фоне референсных показателей тиреоидных гормонов (свТ3 — 3,13 пмоль/л, свТ4 — 9,82 пмоль/л) и положительных АТ к ТПО 301,05 МЕ/мл. Доза левотироксина натрия увеличена до 150 мкг/сутки с последующим контролем ТТГ через 2 месяца. Также обнаружена незначительная гиперпролактинемия до 612,3 мЕд/л на фоне отсутствия признаков гипогонадизма (общий тестостерон — 15 нмоль/л). ИФР-1 197,9 нг/мл и СТГ 0,44 нг/мл — в пределах референсных значений. Согласно результатам МРТ головного мозга с к/у, были выявлены очаговые изменения структуры гипофиза, соответствующие остаточным явлениям кисты кармана Ратке, данных за наличие аденомы гипофиза не получено. Выявленное повышение пролактина объяснялось декомпенсацией гипотиреоза, медикаментозная терапия аналогами дофамина пациенту не назначалась. По поводу хронической крапивницы пациент консультирован дерматологом, выявлен факт чрезмерного приема морепродуктов, даны рекомендации.

При ежегодном динамическом контроле ТТГ составил 39 мМЕ/л при свТ4 — 11,8 пмоль/л и свТ3 — 3,6 пмоль/л на фоне приема левотироксина натрия в дозе 150 мкг/сутки. Даны рекомендации по правилам приема левотироксина натрия с сохранением прежней дозировки.

В 35 лет у пациента по результатам лабораторного исследования отмечена отрицательная динамика в виде увеличения уровня ТТГ до 97,6 мМЕ/л на фоне нормальных показателей свТ4 — 11,1 пмоль/л и свТ3 — 4,8 пмоль/л, и положительных АТ-ТПО 233,6 МЕ/мл. Правила приема препарата соблюдены. Отмечалось по-прежнему повышение общего пролактина до 474,25 мЕд/л, тестостерон — 14,05 нмоль/л, ЛГ 3 мМе/мл, ФСГ — 1,99 мМЕ/мл, эстрадиол — 36,49 пг/мл, ГСПГ 18,4 нмоль/л.

Учитывая длительный анамнез сохраняющейся субкомпенсации первичного гипотиреоза на фоне заместительной терапии левотироксином натрия в отсутствие признаков мальабсорбции, некомплаентности пациента, заподозрен лабораторный феномен макро-ТТГ (табл. 1).

При применении дополнительных методов обследования (преципитация с полиэтиленгликолем (ПЭГ)) уровень биоактивного ТТГ составил 10,4 мМЕ/л из общей фракции ТТГ — 50,1 мМЕ/л, также был исследован общий пролактин — 514,6 мЕд/л, из которого биоактивная фракция составила 146,8 мЕд/л, что характерно для феномена макропролактинемии (концентрация макропролактина в сыворотке крови более 60%) (рис. 1). Таким образом, у пациента с первичным гипотиреозом в исходе АИТ, медикаментозной субкомпенсацией подтверждено наличие сочетания феноменов макро-ТТГ и макропролактинемии, в связи с чем терапия агонистами дофамина не показана, рекомендовано увеличение дозы левотироксина натрия до 175 мкг/сут, под контролем биоактивного ТТГ.

**Table:** Таблица 1. Лабораторные показатели и терапия у пациента в динамике

Год	2015	2017	05.2021	08.2021	04.2022	06.2023	06.2023
	Результаты	
ТТГ, мМЕ/л	159	0,42	94,2	58,8	39	97,6	50,1
Биоактивный ТТГ, мМЕ/л							10,35
свТ4, пмоль/л	9,9	33,1	8,4	9,82	11,8	11,1	
свТ3, пмоль/л			2,6	3,13	3,6	4,8	
АТ к ТПО, МЕ/мл			210	301,05		233,6	
АТ к ТГ, МЕ/мл			4842				
Доза левотироксина натрия, мкг/сут	Инициация — 150	Редукция со 150 до 100	100	Увеличение со 100 до 150	150	150	
ПРЛ, мЕд/л	842	19,94	27,65	612,3	404,25	474,25	514,6
ПРЛ биоактивный,мЕд/мл							146,8
Доза каберголина	0,5 мг 1 раз в 10 дней	0,5 мг 1 раз в 10 дней (нерегулярно)	0,5 мг 1 раз в 10 дней (нерегулярно)	-	-	-	

**Figure:**
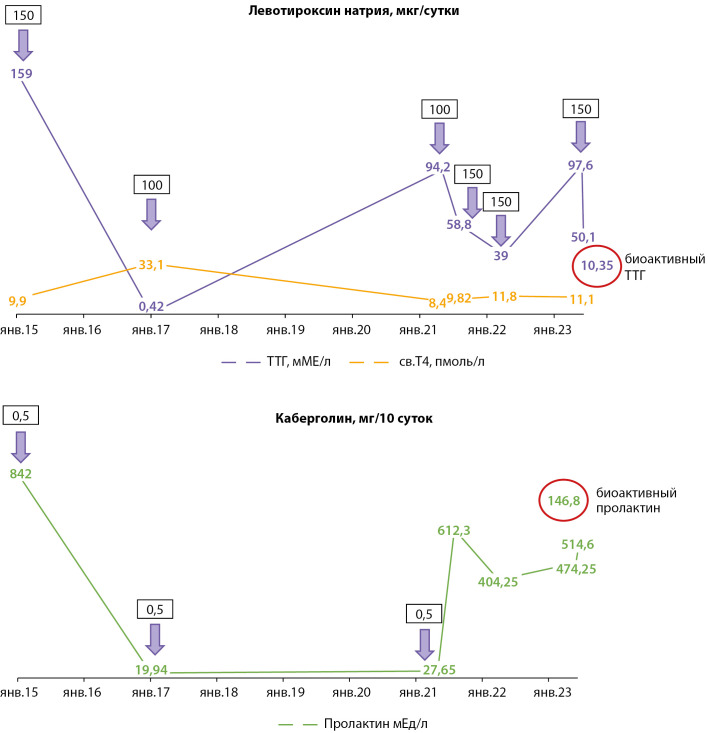
Рисунок 1. Динамика гормональных показателей.

## ОБСУЖДЕНИЕ

Согласно проведенному анализу научных баз данных eLibrary, Cyberleninka и National Library of Medicine, наш клинический случай является первым, описывающим одновременное выявление феноменов макро-ТТГ и макропролактинемии.

Макроизоформы гормонов, получившие дополнительное название в литературе ”incidentalormones” [22], могут быть подводными камнями в практике врача-эндокринолога ввиду их редкой встречаемости. Данные комплексы, как правило, являются гормональными соединениями с пониженной биологической активностью по отношению к их гомологичным рецепторам [23]. Несмотря на высокую чувствительность и специфичность иммунологических тестов, определяющих гормональные показатели крови, могут возникать различные аналитические «вмешательства», связанные с существованием аутоантител, гетерофильных антител и даже антител к животным, приводящих к ложноположительным или ложнотрицательным результатам. Так, из-за больших молекулярных размеров иммунных комплексов их почечный клиренс достаточно затруднителен, что приводит к более продолжительной циркуляции макроизоформ и, как следствие, повышенным показателям общих фракций гормонов. Таким образом, даже с развитием иммунологических тестов, аналитические ошибки, возникающие в результате неспецифических или перекрестных реакций, все еще не полностью предотвратимы.

В отношении причин развития феноменов, связанных с макроизоформами гормонов, проведено множество исследований. Как правило, появление аутоантител ассоциировалось с сопутствующими аутоиммунными заболеваниями щитовидной железы, в частности с АИТ [17][18][24].

В 2005 г. Hattori N. et al., изучая патогенез феномена макропролактинемии, предположили, что стимулирование антигенной активности связано с неадекватным фосфорилированием молекулы пролактина, что, вероятно, расценивается иммунной системой в качестве чужеродного агента [25]. А в другом своем исследовании предположили, что повышенные уровни матриксной металлопротеиназы у пациентов с ревматоидным артритом могут приводить к посттрансляционной модификации молекулы ПРЛ в виде образования новых эпитопов на ее поверхности, которые в последующем приводят к выработке аутоантител к ПРЛ [26].

Согласно исследованию Hattori N. et al. 2014 г., у 6 пациентов с макро-ТТГ был диагностирован АИТ или ДТЗ, что также подтверждает аутоиммунную природу феномена [27]. Интересно, что в исследовании Hattori N. et al. 2017 г., пациенты с феноменом макро-ТТГ также имели другие аутоиммунные заболевания — системную красную волчанку, ревматоидный артрит и системный склероз, а некоторые из этих пациентов получали терапию глюкокортикостероидами или препаратами интерферона. Поэтому предполагается, что системные иммунные нарушения могут играть ключевую роль в развитии аутоиммунной агрессии к гормональным соединениям. Так, на основании исследования антител к ТТГ [28] была высказана гипотеза как о нарушении иммунной толерантности организма, так и изменении антигенности гормонов в процессе естественного старения, способствующих выработке аутоантител.

В литературе приводятся данные о существовании не связанных с IgG макромолекул на примере макропролактинемии, однако проведенные исследования доказывают, что не-IgG опосредованные комплексы составляют минимальную часть макроизоформ [29].

Таким образом, основным патогенетическим звеном остается аутоиммунная этиология, что подтверждается нашим клиническим случаем.

## ЗАКЛЮЧЕНИЕ

Макроизоформы гормонов встречаются в основном в виде комплекса гормона с IgG, который блокирует сайты связывания, тем самым инактивируя исследуемое вещество. Клиницист должен обращать внимание на лабораторное повышение уровней гормонов при нехарактерной для этого клинической картине, особенно у пациентов с аутоиммунными заболеваниями в анамнезе. Таким образом, присутствие макроизоформ гормонов часто ведет к ошибочным заключениям при лабораторных исследованиях и может привести к неправильному диагнозу с избыточной терапией. В свою очередь специалист должен распознавать и исследовать возможные аналитические ошибки при выполнении лабораторной диагностики. Врачу необходимо сопоставлять повышенные уровни гормонов в совокупности с клинической картиной, что поможет правильно определить тактику ведения пациента.

## Дополнительная информация

Источники финансирования. Выполнено в рамках гранта Министерства образования и науки, соглашение № 075–15–2022–310 от 20.04.2022.

Конфликт интересов. Авторы декларируют отсутствие явных и потенциальных конфликтов интересов, связанных с содержанием настоящей статьи.

Участие авторов. Все авторы одобрили финальную версию статьи перед публикацией, выразили согласие нести ответственность за все аспекты работы, подразумевающую надлежащее изучение и решение вопросов, связанных с точностью или добросовестностью любой части работы.

Согласие пациента. Пациентка добровольно подписала информированное согласие на публикацию персональной медицинской информации в обезличенной форме в журнале «Проблемы эндокринологии».
